# Factors associated with return of spontaneous circulation after out-of-hospital cardiac arrest in Poland: a one-year retrospective study

**DOI:** 10.1186/s12872-020-01571-5

**Published:** 2020-06-12

**Authors:** Michał Czapla, Marzena Zielińska, Anna Kubica-Cielińska, Dorota Diakowska, Tom Quinn, Piotr Karniej

**Affiliations:** 1 Department of Public Health, Faculty of Health Sciences, Wroclaw Medical University, Wroclaw, Poland; 2 Department of Anaesthesiology and Intensive Therapy, Faculty of Medicine, Wroclaw Medical University, Wroclaw, Poland; 3 Department of Nervous System Diseases, Faculty of Health Sciences, Wroclaw Medical University, Wroclaw, Poland; 4 Faculty of Health, Social Care and Education Kingston University and St George’s, University of London, London, UK

**Keywords:** Out-of-hospital cardiac arrest, Cardiopulmonary resuscitation, Emergency medical services, Prehospital emergency care

## Abstract

**Background:**

Out-of-hospital cardiac arrest (OHCA) is a common reason for calls for intervention by emergency medical teams (EMTs) in Poland. Regardless of the mechanism, OHCA is a state in which the chance of survival is dependent on rapid action from bystanders and responding health professionals in emergency medical services (EMS). We aimed to identify factors associated with return of spontaneous circulation (ROSC).

**Methods:**

The medical records of 2137 EMS responses to OHCA in the city of Wroclaw, Poland between July 2017 and June 2018 were analyzed.

**Results:**

The OHCA incidence rate for the year studied was 102 cases per 100,000 inhabitants. EMS were called to 2317 OHCA events of which 1167 (50.4%) did not have resuscitation attempted on EMS arrival. The difference between the number of successful and failed cardiopulmonary resuscitations (CPRs) was statistically significant (*p* < 0.001). Of 1150 patients in whom resuscitation was attempted, ROSC was achieved in 250 (27.8%). Rate of ROSC was significantly higher when CPR was initiated by bystanders (*p* < 0.001). Patients presenting with asystole or pulseless electrical activity (PEA) had a higher risk of CPR failure (86%) than those with ventricular fibrillation/ventricular tachycardia (VF/VT). Patients with VF/VT had a higher chance of ROSC (OR 2.68, 1.86–3.85) than those with asystole (*p* < 0.001). The chance of ROSC was 1.78 times higher when the event occurred in a public place (*p* < 0.001).

**Conclusions:**

The factors associated with ROSC were occurrence in a public place, CPR initiation by witnesses, and presence of a shockable rhythm. Gender, age, and the type of EMT did not influence ROSC. Low bystander CPR rates reinforce the need for further efforts to train the public in CPR.

## Background

Out-of-hospital cardiac arrest (OHCA) is a common reason for calls to emergency medical services (EMS) [1]. In the European Union, 300,000 to 700,000 cases of OHCA are recorded every year [2] with reported survival 8–10% [3]. Survival could be improved if more witnesses to the event undertake cardiopulmonary resuscitation (CPR) [4, 5].

The immediate initiation of CPR by event witnesses can increase OHCA survival rates fourfold. Chances of successful outcome diminish by 7–10% for every minute without effective cardiopulmonary resuscitation (CPR) [6]. The actions of bystanders, and rapid response from EMS, are therefore critical. However, the incidence of CPR initiation by bystanders remains low, due to factors including fear of infectious diseases, aversion to mouth-to-mouth ventilation, high-stress levels, and, most important, a lack of knowledge about performing CPR [7].

For this reason, guidelines published by the European Resuscitation Council emphasize the importance of raising public awareness and developing first aid skills of people, including CPR [2]. In Poland, the average EMS response time to a patient is 8 min in urban areas and 15 min in rural areas, which often makes it difficult or even impossible to restore efficient circulation without deep ischemic brain consequences [8].

The aim of this study was to retrospectively analyze factors associated with ROSC in OHCA patients.

## Methods

### Study design and setting

A retrospective analysis was performed on the EMS records of patients with OHCA in the city of Wroclaw, Poland and the surrounding districts from July 2017 to June 2018. The assessment was carried out using the information on medical rescue procedure cards routinely administered by EMS.

Wroclaw and its surrounding districts have more than one million inhabitants. Emergency prehospital care is provided by a single EMS system. EMS Rapid Reaction Forces are organized into 15 substations covering 42 emergency medical teams (EMTs), of which 13 are specialized EMTs (S-EMTs) comprising at least three persons, including a doctor and a medical nurse or paramedic, and 29 are basic EMTs (B-EMTs) comprising at least two persons qualified to perform medical rescue services, including a medical nurse or paramedic,

### Study population

We analyzed 2.317 EMS records, of which 1.167 (50.4%) were rejected because resuscitation was not attempted by EMS (e.g. because the patients died before EMS arrival). Further examination was carried out on 1.150 cards (49.6%) that documented cases in which the resuscitation was attempted by EMS. However, 158 among them did not have the information about the mechanism of cardiac arrest and were excluded from the further analysis.

We analyzed the factors associated with achieving ROSC.

Demographic factors such as age and gender, the circumstances of OHCA, the presence of event witnesses, and CPR initiation were included in the analysis We also assessed whether ROSC was associated with type of EMT (B-EMT or S-EMT) attending. We also examined whether the OHCA mechanism—that is, shockable rhythm, i.e. ventricular fibrillation/ventricular tachycardia (VF/VT) or non-shockable rhythm, i.e. asystole or pulseless electrical activity (PEA)— were associated with ROSC.

The next stage of the study was to examine which of the above factors showed the strongest correlation with ROSC in the group where this was achieved.

### Ethical considerations

This study was approved by the independent Bioethics Committee of the Wroclaw Medical University (decision no. KB–604/2019). The study was carried out in accordance with the tenets of the Declaration of Helsinki and recommendations of good clinical practice. For reporting, the Strengthening the Reporting of Observational Studies in Epidemiology (STROBE) guidelines were followed.

### Statistical analyses

Statistical analysis was performed using Statistica 13 (Tibco Inc., USA). For the arithmetic means, medians, standard deviations, quartiles, and ranges of variation (extreme values) were calculated. For the categorical variables, the frequencies of occurrence (percentages) were calculated. The type of distribution of the numerical variables was identified using the Shapiro-Wilk test. The differences between the groups were determined using the parametric Student’s t-test for independent variables or non-parametric Mann-Whitney U-test depending on the test assumptions. The comparison between the categorical variables was carried out using chi-square tests. The impact of the variables on ROSC was assessed with logistic regression analysis. The multi-factor analysis was performed with use of a stepwise logistic regression model with a forward-entry stepping algorithm; variables with a *P* value of ≤0.05 on single-factor model were entered in the model. Multivariate analysis includes the following variables: Age, Gender, CPR initiated by witness, ECG, Public place, Type of EMT. The results were considered statistically significant if the *p*-value is *p* < 0.05.

## Results

**Fig. 1:**
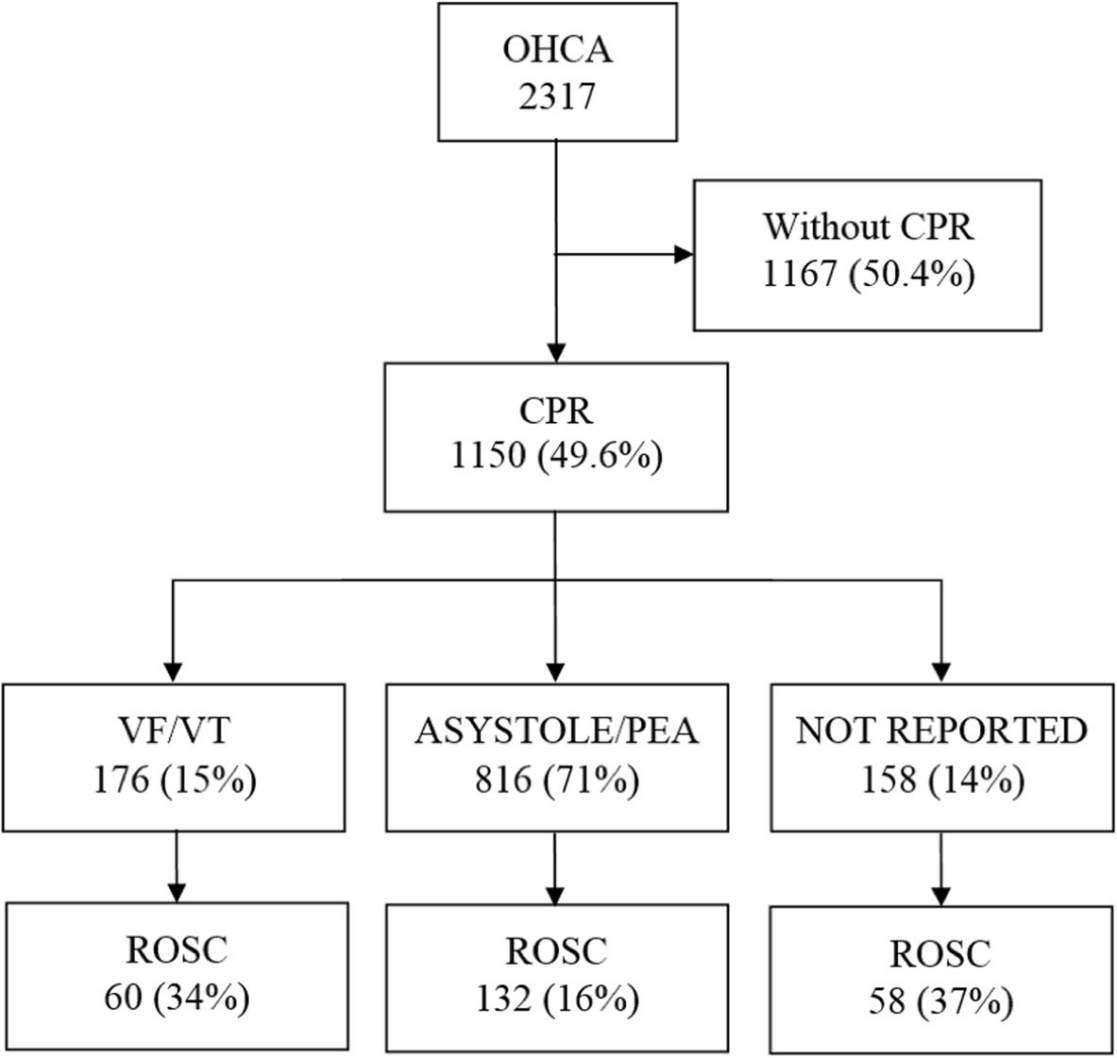
The main data of out-of-hospital cardiac arrest (OHCA). *Abbreviations:* CPR: cardiopulmonary resuscitation; VF: ventricular fibrillation; VT: ventricular tachycardia; PEA: pulseless electrical activity; ROSC: return of spontaneous circulation

The OHCA incidence rate for the year studied was 102 cases per 100,000 inhabitants. Between July 2017 and June 2018, EMTs were called 2.317 times to OHCA cases. Figure 1 presents data on the OHCA cases examined in this study. For 1167 (50.4%) of the calls, CPR was not performed by EMS. 1150 cases where CPR was undertaken, ROSC was achieved in 250 (27.8%).

Table 1 presents the characteristics of the group in which CPR was performed, according to whether ROSC was achieved. The group includes only those whose OHCA mechanism was reported. A total of 992 people was examined, of whom 192 (19.5%) had ROSC and 800 (80.5%) did not. 158 people were excluded from further analysis due to missing information on presenting rhythm. The mean age was 65.5 years (SD = 17.9) for patients with ROSC and 67.7 years (SD = 17.7) for those without ROSC. The age difference between these groups was not statistically significant. Significantly more cases of ROSC were observed in the group where bystander CPR was initiated (21% vs 9%) prior to EMS arrival (*p* < 0.001). In 78% cases with ROSC and 86% without ROSC, there was no record by EMTs regarding who initiated CPR.

**Table 1:** Comparison of selected variables characterizing the study group depending on ROCS

	ROSC				*p*-value*
	Yes (*n* = 192)	%	No (*n* = 800)	%	
Age (years)					
x̅	65.5		67.7		=0.14
Min	4.0		1.0		
Max	99.0		104.0		
SD	17.9		17.7		
Gender					
Women	65	34	246	31	=0.41
Men	127	66	554	69	
Transfer to hospital during CPR					
Yes	0	0	8	1	=0.44
No	192	100	792	99	
CPR initiated by witness					
No	40	21	71	9	< 0.001
Yes	3	2	5	1	
Not reported	149	78	724	91	
ECG					
VF/VT	60	31	116	15	< 0.001
ASYS/PEA	132	69	684	86	
Public place					
Yes	70	37	195	24	0.001
No	122	63	605	76	
Type of EMT					
B-EMT	79	41	375	47	=0.15
S-EMT	113	59	425	53	
Arrival time of EMT (minutes)					
≤8	78	41	312	39	=0.68
> 8	114	59	488	61	
Arrival time of EMT (minutes)					
x̅	16.9		15.7		=0.35
Min	1		0		
Max	85		104		
SD	15.8		15.8		

*test χ^2^

*Abbreviations: x̅* mean, *Min* minimum value, *Max* maximum value, *SD* standard deviation, *n* number of participants, *%* percent, *ROSC* return of spontaneous circulation, *CPR* cardiopulmonary resuscitation, *ECG* electrocardiography, *EMT* emergency medical team, *B-EMT* basic emergency medical team, *S-EMT: EMT* specialist emergency medical team, *VF* ventricular fibrillation, *VT* ventricular tachycardia, *ASYS* asystolia, *PEA* pulseless electrical activity

In 684 patients whose mechanism of cardiac arrest was asystole or PEA, ROSC was not achieved; this group constituted 86% of all subjects without ROSC. Among those who had ROSC, 132 (69%) had asystole or PEA documented as the initial rhythm (*p* < 0.001). Moreover, the occurrence of ROSC varied according to where the event occurred. Among those who had an OHCA in a public place, ROSC occurred in 70 cases (37% of those with ROSC) and was not achieved in 195 cases (24%; *p* = 0.001). There was no difference in the effectiveness of CPR according to the type of EMT that arrived at the event location and the time from the call to the initiation of CPR by the EMT (*p* = 0.15) (Table 1).

Single-factor logistic regression model showed the impact of the OHCA mechanism and the event location on ROSC (Table 2).

**Table 2:** Evaluation of the impact of selected variables on the ROSC (single-factor model)

ROSC (probability modeling: yes)						
Variables	B	SE	*p*-value	OR	95% CI (lower)	95% CI (upper)
Age	−0.01	0.01	0.15	0.99	0.98	1.00
Gender						
Women	Ref.					
Men	−0.14	0.17	0.41	0.87	0.62	1.21
CPR initiated by witness						
No	Ref.					
Yes	−0.06	0.76	0.93	0.94	0.21	4.14
Not reported	−1.07	0.73	0.15	0.34	0.08	1.45
ECG						
ASYS/PEA	Ref.					
VF/VT	0.99	0.19	< 0.001	2.68	1.86	3.85
Public place						
No	Ref.					
Yes	0.58	0.17	< 0.001	1.78	1.27	2.49
Type of EMT						
B-EMT	Ref.					
S-EMT	0.23	0.16	0.15	1.26	0.92	1.74
Arrival time of EMT (minutes)						
≤8	Ref.					
> 8	−0.07	0.16	0.68	0.93	0.68	1.13
Arrival time of EMT (minutes)	0.00	0.01	0.35	1.00	0.99	1.01

*Abbreviations: OR* Odds Ratio, *CI* Confidence Interval, *SE* Standard Error, *B* Regression Coefficient, *ROSC* return of spontaneous circulation, *ECG* electrocardiography, *EMT* emergency medical team, *B-EMT* basic emergency medical team, *S-EMT: EMT* specialist emergency medical team, *VF* ventricular fibrillation, *VT* ventricular tachycardia, *ASYS* asystolia, *PEA* pulseless electrical activity

The likelihood of ROSC was higher in patients with VF/VT than in those with asystole or PEA (OR 2.68, 1.86–3.85 *p* < 0.001). An additional factor enhancing the chance of ROSC was the event location. The likelihood of ROSC was almost twice as high for OHCA that occurred in a public place than in a non-public place, such as at home (OR 1.78, 1.27–2.49 *p* < 0.001). These results were confirmed in a multifactor model (Table 3).

**Table 3:** Evaluation of the influence of selected variables on the ROSC (multi-factor model)

ROSC (probability modeling: yes)						
Variables	B	SE	*p*-value	OR	95% CI (lower)	95% CI (upper)
ECG						
ASYS/PEA	Ref.					
VF/VT	0.92	0.19	< 0.001	2.52	1.74	3.63
Public place						
No	Ref.					
Yes	0.43	0.21	0.045	1.53	1.01	2.32

Hosmer-Lemeshow’s test: *p* = 0.24

Multivariate analysis includes the following variables: Age, Gender, CPR initiated by witness, ECG, Public place, Type of EMT

*Abbreviations: OR* Odds Ratio, *CI* Confidence Interval, *SE* Standard Error, *B* Regression Coefficient, *ECG* electrocardiography, *VF* ventricular fibrillation, *VT* ventricular tachycardia, *ASYST* asystolia, *PEA* pulseless electrical activity

Table 4 presents a comparison of bystander CPR according to the patient’s gender and the event location. In public places, event bystander CPR was more frequently initiated when the patient was male (52%) compared to when the patient was female (28%; *p* = 0.032).

**Table 4:** Comparison of initiations of CPR by witnesses according to their gender and place of event

	CPR initiated by witness Yes			CPR initiated by witness No		
	Public place		*p*-value	Public place		*p*-value
	Yes	No		Yes	No	
Gender						
Women	7	18	=0.032	0	3	=0.90
%	28%	72%		0%	100%	
Men	45	41		3	2	
%	52%	48%		60%	40%	

*χ^2^ test

*Abbreviations:* n: number of participants; %: percent

## Discussion

The OHCA incidence in the city of Wroclaw for the studied year was 102 for every 100,000 inhabitants. This is lower than the incidence of 123 for every 100,000 inhabitants in a similar study by Daniels et al. [9] in the district of Udine in Italy. It is also lower than the incidence in Vienna, which, according to Nürnberger et al. [10], was 207 for every 100,000 inhabitants. In other part of Europe about 55–113 per 100,000 inhabitants a year are affected [11]. The reason for these differences is not entirely apparent, but the incidence is likely affected by many factors, including the population characteristics and organization of EMS of the studied region. The average age of the patients in our study was 65.5 years and 67.7 years for patients with and without ROSC, respectively. This is slightly lower than in other studies conducted in the European Union, where the average age of OHCA patients was between 65 and 75 years [12].

The literature indicates that the majority of OHCA occurs in residential settings, as was the case in our study, this was also the case, whereas 73% occurred at home. This is comparable with reports from Japan (84%), Singapore (70%) [13], and South Korea (65–69%) [14]. An important determinant of survival was the place where the OHCA occurred. When OHCA occurred in a public place, the chances of ROSC were almost twice as high as when the incident occurred at home. This finding is similar to that of a study in Detroit [15]. It should be considered that in a public place, the probability of the presence of a witness of cardiac arrest who has CPR knowledge and skills is higher.

Many studies have underlined the importance of bystander CPR, yet rates of bystander CPR remain low, ranging from 13 to 35% [9, 16, 17]. In our study, bystander CPR was recorded in 21% of patients who achieved ROSC. Barriers to performing bystander CPR are well documented in the literature, with lack of knowledge or fear of harming the victim, feeling of fear or concern about getting an infectious disease [18, 19]. Given that shortening the ‘no flow’ time of brain ischemia improves the patient’s chances of a good neurological outcome, there is a need for accessible, frequent, and repeated courses on CPR. Where bystanders are deterred because of fears of infection or for aesthetic reasons, from providing mouth-to-mouth ventilation, compression – only CPR has been suggested as an option [20].

Of note is the finding that witnesses are much more willing to give CPR to men than to women. Many studies have confirmed that women are less likely to receive bystander CPR even when OHCA occurs in the presence of witnesses [21]. In the study by Blewer et al. [22] of 19.331 OHCA cases, when the incident occurred in a public place, 39% of women and 45% of men received help—higher than the results obtained in the present study (28 and 53%, respectively).

Another factor that increased the chances of ROSC was the mechanism of OHCA. When OHCA occurred in a shockable rhythm, the likelihood of ROSC increased 2.68 times (*p* < 0.001). This finding is confirmed by the literature. A shockable rhythm is considered a beneficial prognostic factor [17]. In a cohort study analyzing OHCA in 27 European countries, the prevalence of OHCA in a shockable rhythm was 22%, compared to 15% in our study. However, of the ROSC patients we analyzed, as many as 34% had VT/VF rhythms as the cause of OHCA; in other studies, 13 to 54% of the patients with ROSC showed shockable rhythms [9, 16, 23, 24].

In our study, there were no statistically significant differences in ROSC according to gender, age, or the type of EMT. The probability of the presence of a witness of cardiac arrest who has CPR knowledge and skills is higher. There was also no statistically significant difference in ROSC according to the arrival time of the EMT (*p* = 0.68). However, other studies have drawn attention to the need for a rapid EMS response. Response time ≤ 7.5 min may lead to favorable neurological outcomes in OHCA patients [25]. Goto et al. [26] reported that the upper limits of EMS response times associated with improved 1-month neurologically intact survival were 13 min when a witness started CPR and provided defibrillation, and 11 min CPR was initiated without defibrillation. On the other hand, Bürger et al. [27] reported that rapid ambulance response is associated with a higher rate of survival from OHCA with good neurological outcome.

Some studies have pointed out that the presence of a doctor supervising the work of qualified paramedics improves the effectiveness of CPR and increases the chances of ROSC [17, 28]. However, our results did not show statistically significant differences in the success rate of CPR depending on the type of EMT—a finding that is consistent with those of other studies [29, 30]. Fullerton et al. [29] and Kupari et al. [30] did not find any differences in ROSC according to the presence of a doctor in the EMT. It is worth emphasizing that despite the variety of models of EMS provision in different countries, most of the studies evaluating the factors affecting ROSC did not take this factor into account [31–33]. Another factor which could influence outcomes from OHCA is the individual’s cardiorespiratory fitness level of the patients before cardiac arrest. According to Laukkanen et al. [34], it plays an essential role as the risk factor of VT/VF, arrhythmias (AF, atrial fibrillation), and sudden cardiac death (SCD) [35, 36]. Since such data are not recorded by EMTs, we were unable to take into account in our analyses.

### Limitations

Our study has several limitations, principally related to the EMS documentation available to us for analysis. Firstly, there was a lack of information on the OHCA mechanism in the electrocardiographic examination results. Secondly, we did not have information on bystander CPR in 78% of patients in the ROSC group and 91% in the no-ROSC group, as this was not recorded by EMTs. Thirdly, documentation on the presence of witnesses to the OHCA event or about bystander CPR was limited. Fourthly, EMS documentation lacked valuable information such as other factors influencing outcomes, such as use of adrenaline, duration of CPR, ‘no flow’ time from the arrest to CPR starting, number of shocks delivered etc. In many documents, it was only noted that CPR was carried out in accordance with the guidelines of the European Resuscitation Council. Fifth, because of restrictions on access to personal data due to the anonymity of EMS records in the study setting, it was not possible to follow up survivors to assess important patient-focused outcomes such as neurological and functional status, survival to and beyond discharge, and quality of life. Future studies need to be designed to address these important outcomes, and to improve the documentation of OHCA within the EMS system.

## Conclusions

The factors that increased the likelihood of ROSC were the public location of the event, the initiation of CPR by witnesses, and the shockable rhythm of the OHCA episode. Factors not associated with ROSC were gender, age, and type of EMT. The analysis confirmed the low incidence of CPR by the event witnesses, which further supports the need to continue intensive first aid training among as many different social groups as possible.

## Data Availability

The datasets used and/or analyzed during the current study are available from the corresponding author on reasonable request.

## Abbreviations

AF: Atrial fibrillation
B-EMT: Basic emergency medical team
CPR: Cardiopulmonary resuscitation
EMS: Emergency medical services
EMT: Emergency medical team
OHCA: Out-of-hospital cardiac arrest
PEA: Pulseless electrical activity
ROSC: Return of spontaneous circulation
S-EMT: Specialized emergency medical team
STROBE: Strengthening the reporting of observational studies in epidemiology
SCD: Sudden cardiac death
VF: Ventricular fibrillation
VT: Ventricular tachycardia
